# Paediatric extra-axial myxopapillary ependymoma: what to expect? Case report and literature review

**DOI:** 10.3389/fonc.2025.1519842

**Published:** 2025-01-30

**Authors:** Roberto Lo Piccolo, Maria Chiara Cianci, Iacopo Sardi, Marco Di Nicola, Anna M. Buccoliero, Chiara Caporalini, Antonino Morabito

**Affiliations:** ^1^ Azienda Ospedaliera Universitaria Meyer Istituto di Ricerca e Cura a carattere Scientifico (IRCCS) - Firenze, Florence, Italy; ^2^ Department of Pediatric Surgery, Istituto di Ricerca e Cura a carattere Scientifico (IRCCS) Meyer Children’s Hospital, Florence, Italy; ^3^ Neuro-Oncology Unit, Meyer Children’s Hospital, Florence, Italy; ^4^ Pathology Unit, Meyer Children’s Hospital, Florence, Italy; ^5^ Department of Neuroscience, Psychology, Drugs and Child Health Area, School of Psychology, University of Florence, Florence, Italy

**Keywords:** paediatric, sacral soft tissue lesion, case report, myxopapillary ependymoma, extra-axial ependymomas

## Abstract

Extra-axial ependymomas are rare tumours, and myxopapillary ependymoma (MPE) is the most common subtype in children, often misdiagnosed as other sacral lesions. MPEs are considered low-grade tumours, but relapse with distant metastasis is frequent. Therefore, therefore a proper diagnosis and subsequent follow-up are warranted. The current report presents a case of extra-axial MPE in a paediatric patient who presented with an indolent sacral mass and underwent surgical resection, along with a review of the literature. The aim was to highlight the importance of diagnostic suspicion in differential diagnosis of sacral soft-tissue masses

## Introduction

Ependymomas arise from the ependymal cells lining the ventricular system of the central nervous system (CNS) and represent the third most common CNS tumours in the paediatric population (1).

Ependymal tumours are classified in accordance with the WHO Classification of Tumours of the Central Nervous Systems and according to ICD-O-3 histology/behaviour codes (2). The latter classification describes the following: ependymoma (9391/3 cellular ependymoma, clear cell ependymoma, and tanycytic ependymoma, and 9393/3 papillary ependymoma), anaplastic ependymomas (9392/3), myxopapillary ependymoma (MPE) (9394/1), and subependymomas (9383/1) (3).

Up to 90% of ependymomas are intracranial, and they are exceedingly rare outside the CNS; however, of all of the primary CNS neoplasms, ependymomas have the greatest propensity to present in extra-axial sites, as in the mediastinum, lung, ovary, pelvis, perianal area, and sacrococcygeal soft tissues (4–6). MPE is the most frequent subtype in children with extra-axial presentation, typically in the sacral soft tissue, without any connection to the CNS (4). Cimino and colleagues recently analysed that MPEs have an extra-axial localization in a significantly higher proportion of individuals 20 years old or less than in those over 20 years old at the time of diagnosis (p < 0.0001) (6). MPEs usually develop from the ependymal surface of the ventricular system or spinal central canal, while the pathogenesis of sacral soft-tissue MPEs is unclear (7). Studies have suggested that MPEs arise from heterotopic ependymal rests or coccygeal medullary vestiges that act as scaffolds for cell migration during CNS development (8, 9).

MPEs are considered low-grade tumours, classified as grade I or II according to WHO Classification 2021, and present as an indolent slow-growing and well-circumscribed mass, often misdiagnosed as other sacral lesions and rarely disseminating within the CNS (3, 10–12). Nevertheless, metastasis and local recurrence occur in up to 20% of cases, particularly with soft-tissue MPEs, involving the lungs, regional lymph nodes, and liver (5, 10, 13–16). A relapse with distant metastases can occur even after 10–20 years; therefore, accurate management and follow-up are warranted (1, 17).

The aim of the current report was to present a paediatric case of extra-axial MPE and a review of the literature to highlight the importance of diagnostic suspicion in the differential diagnosis of sacral soft-tissue masses.

## Case description

**Figure 1:**
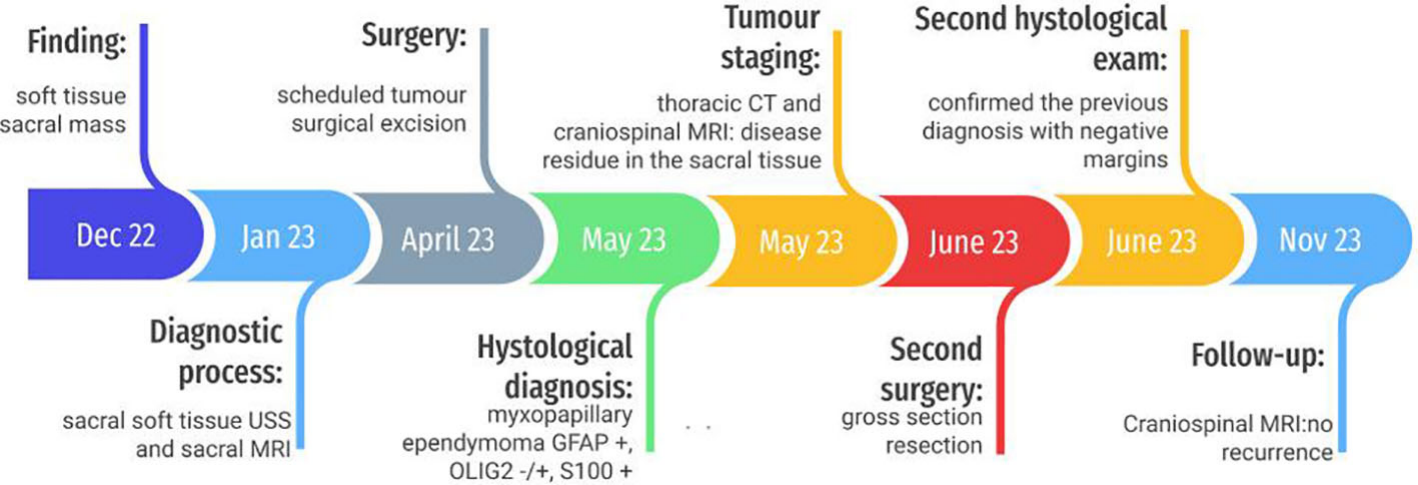
Timeline.

An otherwise healthy 6-year-old boy was admitted to our outpatient clinic for a soft paramedian sacral mass on his left side. He did not report any pain or other symptoms. Familiar history was negative for neoplastic lesions or other pathologies (**Figure 1**).

## Diagnostic assessment, intervention, and outcome

Ultrasound (US) scan was performed, showing non-vascularized solid hypoechoic oval formations with well-defined margins, uneven due to the presence of thin images, located in the subcutaneous adipose tissue and some in contiguity with the posterior profile of the sacrococcygeal vertebrae. There was a suspicion of a neurinoma or ependymoma. Therefore, a magnetic resonance imaging (MRI) of the pelvis was performed: in the subcutaneous of the gluteal region, posteriorly to the sacrum-coccyx, a 16 × 16 × 26 mm left paramedian formation with polylobate margins was recognized. The lesion was in close contact with the posterior margin of the coccyx and the last sacral soma, in the absence of signs of erosion of their cortical profile or other signal alterations, and above it appeared in proximity with the terminal portion of the vertebral canal. Gd-enhanced T1-weighted MRI scans clearly showed a widespread enhancement of the lesion.

**Figure 2:**
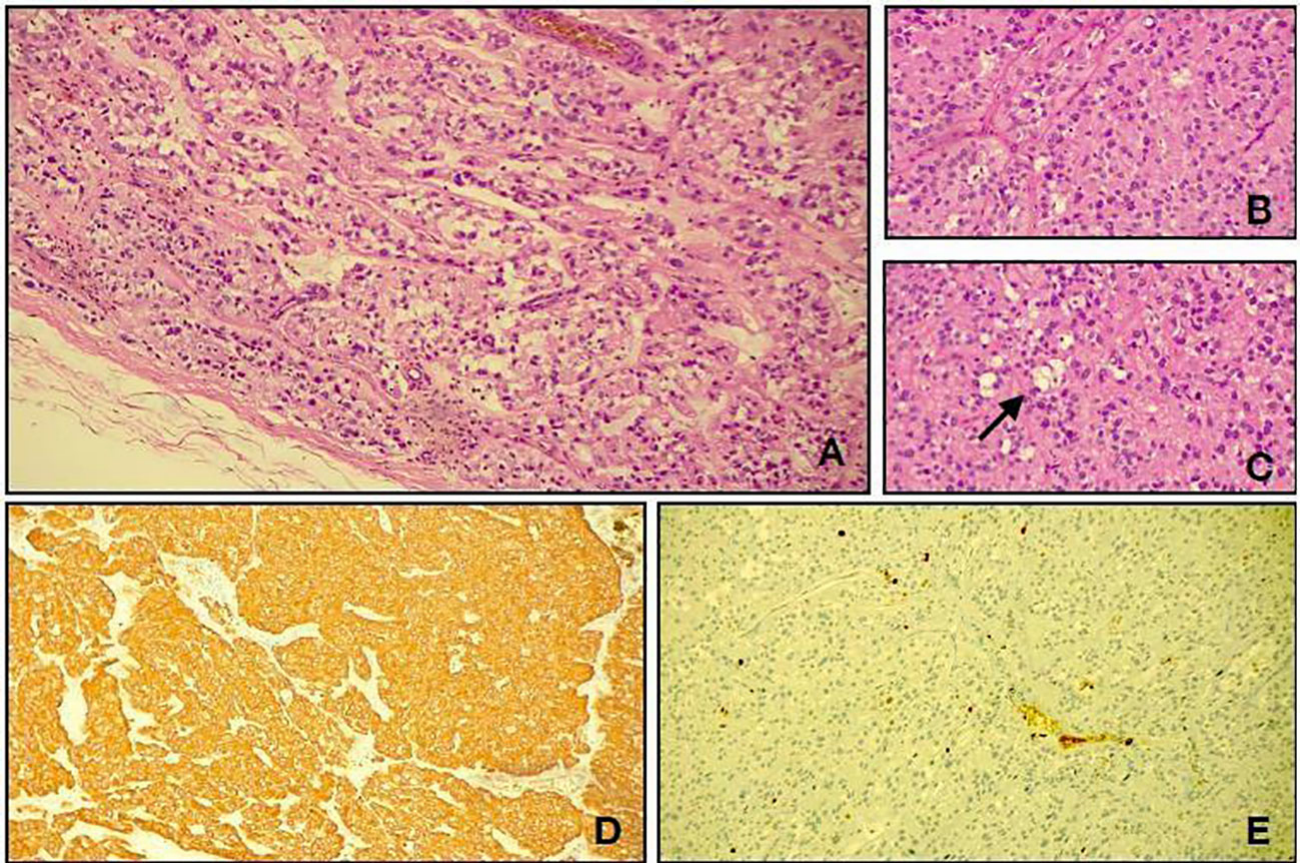
Light microscopy shows a neoplasm characterized by cuboidal to elongated tumour cells arranged around hyalinized fibrovascular cores (**A**, hematoxylin and eosin, original magnification, ×10; **B**, hematoxylin and eosin, original magnification, ×20). A deposition of myxoid material (arrow) was present between the tumour cells and in microcystic spaces (**C**, hematoxylin and eosin, original magnification, ×20). Immunohistological studies showed positive staining for glial fibrillary acidic protein (**D**, GFAP, original magnification, ×10). The Ki67 labelling index (**E**, Ki67, original magnification, ×20).

A surgical resection was performed. No association with nerves or other structures was detected. Histological diagnosis was achieved: myxopapillary ependymoma GFAP 57 +, OLIG2 −/+, and S100 + (WHO grade 2, 2021) (**Figure 2**).

Tumour staging consisted of a thoracic computed tomography (CT), which was negative for metastases, but craniospinal MRI posed the suspicion of disease residue in the sacral tissue (millimetric left parasagittal cystic-like formation, with contrast enhancement).

A second surgery was scheduled, and diamond removal of residual tissue with 10-mm margins was performed, including all material up to the presacral band. The second histological exam confirmed the previous diagnosis with negative margins. Cerebrospinal fluid (CSF) cytology was negative. No adjuvant treatments were requested, and no recurrence or secondary disease was detected. The latest MRI performed 6 months after diagnosis still showed no signs of recurrence; the patient was clinically well.

## Discussion

Extra-axial ependymomas are extremely rare benign tumours in the paediatric population, firstly described by Mallory in 1902 (18). Due to the rarity of this pathology, most cases found in the literature are small series and case reports.

We report a paediatric case of sacral soft-tissue MPE. Moreover, an extensive narrative review of the literature has been performed in order to better define the accurate management of MPEs. A PubMed search was performed with the subsequent keywords: subcutaneous myxopapillary ependymoma AND (child* OR pediatric* OR paediatric). From 1972, a total of 47 papers were found, and of them, 10 were reviews (**Table 1**).

**Table 1:** Subcutaneous paediatric myxopapillary ependymoma.

Reference	MPEs (n)	Preop MRI (Y/N)	Indications for treatment	Associated pathology	Age at intervention	Surgery	MPE’s local infiltration	Adjuvant CT	Adjuvant RT	Follow-up	Alive (n)	Complications	Local recurrence	DNAD	SSM	Distant metastasis	Period between the first diagnosis and diagnosis of recurrence	Treatment of recurrence
Wolff, 1972 (31)	1	N	Suspicion of pilonidal cyst	N	4 y	GTR	N	N	N	20 y	1	0	0	0	0	Inguinal lymph nodes and lung	19 y	GTR
Bale, 1984 (4)	1	–	Growing mass	N	4 y	GTR	N	N	N	20 y	1	0	0	0	0	Inguinal lymph nodes	–	–
Matsuo, 1985 (15)	1	N	Suspicion of pilonidal cyst	N	11 y	GTR	N	N	N	7 m	1	0	0	0	0	0		
Ciraldo, 1986 (34)	5	–	–	N	10, 4 m	GTR	N	N	N	25.6 m	5	0	0	0	0	0		
Murphy, 1987 (35)	1	–	–	N	5 d	GTR	N	N	N	–	1	0	0	0	0	0		
Le Marc’Hadour, 1991 (36)	1	–	–	N	14 y	GTR	N	N	N	2 y	1	0	0	0	0	0		
Botti, 1994 (22)	1	N	Growing mass	N	10 y	GTR	N	N	N	–	1	–	0	0	0	0		
Ihlan, 1998 (20)	1	Y	Growing mass	N	8 y	GTR with coccygectomy	N	N	N	20 m	1	0	0	0	0	0		
Lemberger, 1989 (37)	1	–	–	N	18 m	GTR	N	N	N	–	1	0	0	0	0	Inguinal lymph nodes and lung	–	–
Rao, 2002 (25)	1	N	Growing mass	N	16 m	GTR with coccygectomy	N	1	N	–	1	0	0	0	0	0		
Trobs, 2006 (24)	1	Y	Growing mass	N	9 y	GTR with partial coccygectomy	N	N	N	6 m	1	0	0	0	0	0		
Cimino, 2014 (6)	7	Y	Growing mass	N	7.4 y (3 w-17 y)	GTR	N	N	N	5 y	7	0	0	0	0	0		
Dogan, 2016 (23)	1	Y	Growing mass	N	9 y	GTR	N	N	N	6 m	1	0	0	0	0	0		
Schiavello, 2018 (1)	6	Y	Growing mass	N	10 y (4–16 y)	GTR (1); GTR with coccygectomy (1); STR (4)	Coccyx infiltration (2)	1	1	12.8 y	6	0	2	0	0	Inguinal lymph nodes and sacral vertebrae (1); parauterine region and lung (1)	4 (2–6) y	CT, RT, and myeloablative melphalan (1); GTR followed by CT and RT (1)
Vetrano, 2018 (26)	1	Y	Suspicion of lipoma	Conus lipoma, determining tethered cord	11 m	GTR with conus tabularization and duroplasty	N	N	N	1 y	1	0	0	0	0	0		
Gupta, 2020 (5)	1	N	Suspicion of pilonidal cyst	N	9 y	GTR	N	N	N	18 m	1	0	0	0	0	0		
Johnson, 2023 (7)	1	Y	Discovery of tethered cord with associated lipoma		14 m	GTR with laminectomy for spinal cord	N	N	N	5 y	1	0	0	0	0	0		

Y/N, yes/no; y, year; m, month; w, week; d, day; GTR, gross total resection; STR, sub-total resection; CT, chemotherapy; RT, radiotherapy; MPEs, myxopapillary ependymomas; DNAD, distant neural axis dissemination; SSM, spinal seeding metastasis.

Although we reported only one case, from the analysis of the literature, it emerged that it is in line with the characteristics of the previously described cases. As a matter of fact, sacral soft-tissue MPEs usually present as an indolent slow-growing mass, and they are often preoperatively misdiagnosed as different conditions, such as pilonidal cysts, teratomas, chordomas, lipomas, sweat gland tumours, metastatic masses, neurofibromas, abscesses, and myelocystocele (10). Ependymal cell rests of the sacrococcygeal area also should be included in differential diagnosis, as firstly suggested by Pulitzer (19). These lesions share some histological patterns with MPEs, but they lack neoplastic characteristics, leading to a different prognosis (19).

Different authors reported their experience with misdiagnosed soft-tissue MPEs (especially with pilonidal cysts and sacrococcygeal teratoma), where only histological study leads to the proper diagnosis (15, 20–25). As a matter of fact, MPEs are characterized by glial fibrillary acidic protein (GFAP) immunoreactivity, but CD99 and CD56 reactivity, although not specific, is also frequently reported. Immunolabeling for S-100 protein, anti-vimentin, and anti-keratin antibodies is further mentioned (15, 20).

We described a myxopapillary-type lesion, characterized by mucin and papilla production. However, extra-axial grade II ependymomas and ependymomas with ependymoblastoma differentiation or anaplastic differentiation (with infiltrative growth pattern, atypia, and mitosis) have also been reported (10, 13). Recently, Planas and colleagues described a case of giant cell ependymoma (GCE) in an otherwise healthy 8-year-old girl presenting with a mass in the soft tissue of the sacral region, initially misdiagnosed as a vascular malformation, who underwent sclerotherapy without benefit. Due to the persistent growth of the mass, a complete resection was performed, and the pathological diagnosis confirmed GCE (10). MPEs are often described without connection to the filum terminale or cauda equina, but association with CNS anomalies may also be detected (25). Recently, two authors described rare cases of MPEs, incidentally found within a dermal sinus tract, associated with tethered cord syndrome and lipoma of the filum terminale and with conus lipoma, respectively (7, 26).

In our case, as previously described, it was challenging to distinguish an extra-axial MPE from other sacral soft-tissue masses on radiological imaging due to the non-unique features. However, MRI is considered the best imaging modality for detecting, grading, and staging these lesions (13, 27).

Staging of extra-axial ependymoma is crucial because of the 20% risk of metastasizing to systemic organs and local recurrence, as also reported by Helwig’s case series (10, 13–15, 21). In the case of subcutaneous ependymomas, particularly MPEs, local recurrences are less frequent (25% at 15 years), but distant metastases are more common (1, 28, 29). Rarely, also distant neural axis dissemination (DNAD) and spinal drop metastasis (SDM) are reported, particularly for subcutaneous MPEs that occur at the sacrococcygeal region (11, 16). The disease progresses slowly but steadily, even years after removal of the primary tumour; therefore, long-term follow-up is essential to detect possible distant or locoregional recurrence (1, 10, 17, 30). As a matter of fact, Wolffs firstly reported a case of lung metastasis that occurred approximately 20 years after the resection of the primary tumour (31). However, according to Cimino and colleagues’ analyses, MPE in children is more likely to present in the extramedullary soft tissues of the sacrococcygeal region where its behaviour is more indolent than those tumours arising in the spinal cord (6).

No standardized guidelines are available for the treatment of soft-tissue MPEs; however, gross total removal is the favoured treatment (16). The en-bloc excision of the top of the coccyges has been tested, but no improvement in the prognosis was observed unless the bone was involved (24).

The capsular violation during surgery or an incomplete excision can lead to a high rate of recurrence, approximately 41%; therefore, adjuvant treatment is suggested (1, 13, 32). After gross total resection, the role of radiotherapy (RT) and chemotherapy (CHT) remains controversial. Significantly, the decision-making process should include the patient’s neurological function and parents’ choice (11). Some authors recommended RT to provide control of residual, metastatic, and recurrent MPEs (5, 10–13, 33). However, it has also been reported as a wait-and-see strategy without adjuvant RT (9). We adopted this approach for our patient, who is now in complete remission. CT has been used in patients with recurrent disease refractory to resection and radiation, with some favourable results compared to adult data but with uncertain clinical value (1, 12, 13, 21, 33).

The case we mentioned affirmed that extra-axial MPE is a rare tumour that needs to be included in the differential diagnosis of sacral soft-tissue lesions. The suspicion is crucial to achieve the proper diagnosis and management. During long-term follow-up, the risk of local recurrence and metastasis should always be considered, even after the primary lesion has been treated. Consequently, young patients with sacral soft-tissue MPEs must be brought to specialized paediatric centres that can provide multidisciplinary care.

## Patient perspective

Although the patient needed a second surgery, the patient’s family was satisfied with the treatment received, and the patient was carefully followed up.

## Data Availability

The original contributions presented in the study are included in the article/supplementary material. Further inquiries can be directed to the corresponding author.
